# Association between vegetable, fruit, and flavonoid-rich fruit consumption in midlife and major depressive disorder in later life: the JPHC Saku Mental Health Study

**DOI:** 10.1038/s41398-022-02166-8

**Published:** 2022-09-26

**Authors:** Zui Narita, Shoko Nozaki, Ryo Shikimoto, Hiroaki Hori, Yoshiharu Kim, Masaru Mimura, Shoichiro Tsugane, Norie Sawada

**Affiliations:** 1 Department of Behavioral Medicine, National Institute of Mental Health, National Center of Neurology and Psychiatry, Tokyo, 187-8553 Japan; 2 Department of Neuropsychiatry, Keio University School of Medicine, Tokyo, 160-8582 Japan; 3 Department of Psychiatry, National Hospital Organization Shimofusa Psychiatric Medical Center, Chiba-city, Chiba Japan; 4 Division of Cohort Research, National Cancer Center Institute for Cancer Control, Tokyo, 104-0045 Japan; 5 National Institute of Health and Nutrition, National Institutes of Biomedical Innovation, Health and Nutrition, Tokyo, 162-8636 Japan

**Keywords:** Depression, Human behaviour

## Abstract

We evaluated the association between vegetable and fruit consumption – particularly flavonoid-rich fruits – in mid-life and major depressive disorder (MDD) in later life. We also evaluated the association of nutrients in fruits and vegetables with MDD. Vegetable and fruit consumption and nutrient intake for 1204 individuals were averaged from data obtained in 1995 and 2000. MDD was diagnosed by certified psychiatrists in 2014–2015. Logistic regression was used to examine the odds of MDD according to quintile of vegetable and fruit consumption and quartile of nutrient intake. We fitted two regression models, using hierarchical adjustment for age, sex, employment status, alcohol consumption, current smoking, and physical activity. Bias-corrected and accelerated bootstrap confidence intervals were used to obtain accurate information. In fully adjusted models, the highest quintile of total fruit consumption excluding juice and flavonoid-rich fruit consumption showed decreased odds of MDD compared with the lowest quintile (OR = 0.34, 95% CI = 0.15–0.77; OR = 0.44, 95% CI = 0.20–0.97, respectively). No significant association was found for total vegetables and fruits, total vegetables, or total fruits. No significant association was found for any nutrient. This study provides novel information on the association between MDD and flavonoid-rich fruits.

## Introduction

Mental illness is a significant cause of disability and a major component of the global burden of disease [1, 2]. Mental illness has the longest years lived with disability (YLDs) and the same level of disability-adjusted life-years (DALYs) as cardiovascular and circulatory diseases [3]. Among mental illnesses, depression accounts for the largest portion of the burden in YLDs and DALYs [3]. The prevalence of major depressive disorder (MDD) reportedly ranges from 6.0% in low-income countries to 7.1% in high-income countries [4]. The estimated cost of depression and anxiety to the global economy is US$ 1 trillion per year in lost productivity [5]. Thus, the prevention of depression is crucial to reducing the overall burden of disease [6].

Prospective cohort studies have suggested that vegetables and fruits have a preventive effect on depression [7–10]. A meta-analysis showed an inverse association between vegetable and fruit consumption and the risk of depression [11]. This finding was subsequently validated in a newer meta-analysis [12]. The mechanisms underlying this antidepressive effect might be explained by Vitamin C, Vitamin E, or carotenoids that reduce oxidative stress [13–17]. Of note, however, little effort has been made to examine such effects in cohort studies, particularly using data from Asian participants.

Flavonoids are polyphenolic compounds present in fruits that may have a preventive effect against depression. Suggested mechanisms of this effect include an increase in brain-derived neurotrophic factor levels [18, 19], suppression of oxidative stress [13, 20], and neuroinflammation [21–23]. A recent meta-analysis demonstrated antidepressive effects of flavonoids [24]. Moreover, food-based analyses reported that the consumption of apples, pears, and citruses, which are rich in flavonoids, was associated with lower depression risk [25]. To date, however, little information has appeared on the antidepressive effects of overall flavonoid-rich fruits using population-based data from men and women.

Here, to evaluate the association between flavonoid-rich fruits and MDD, we analyzed data from 1204 participants of the Japan Public Health Center-based Prospective Study. We hypothesized that flavonoid-rich fruit consumption would be inversely associated with a diagnosis of MDD. Further, we also studied vegetables, other fruits, and nutrients contained in fruits and vegetables to determine mechanisms potentially involved in this preventive effect.

## Methods

### Study population

**Fig. 1:**
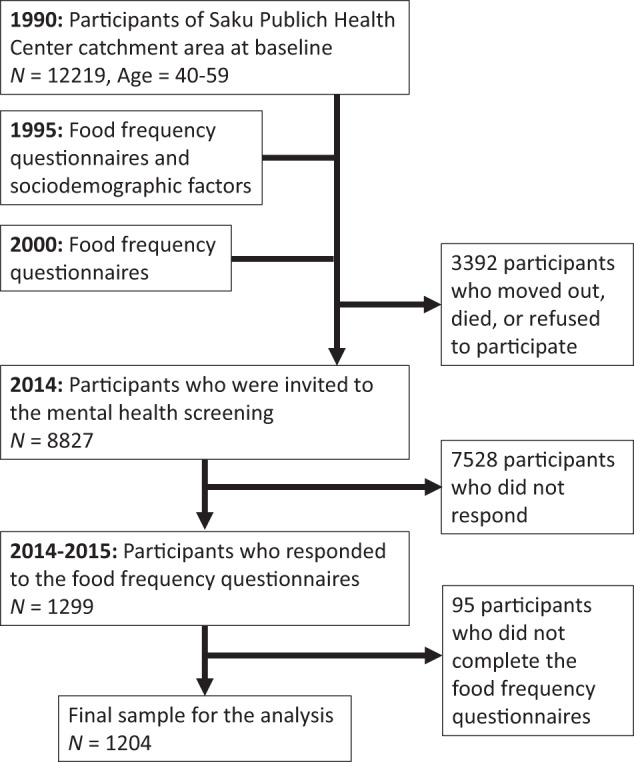
Flow diagram of study population selection. We included 1204 participants in the final sample.

We used data for participants from one region of the Japan Public Health Center-based prospective study (JPHC study) [26]. Among 12219 individuals aged 40–59 years as of 1990 in the Saku Public Health Center catchment area, 8827 were invited to a mental health screening in 2014–2015 after the exclusion of 3392 who moved out, died, or did not respond to questionnaires. Of the 1299 individuals who completed the screening, 1204 (704 women and 500 men) had data from food frequency questionnaires conducted in 1995 and 2000 and were included in the final sample (Fig. 1). All participants provided written informed consent. The present study was approved by the Institutional Review Board of the National Cancer Center and the National Center of Neurology and Psychiatry.

### Diagnosis of major depressive disorder (outcome)

In 2014–2015, certified psychiatrists interviewed the participants and diagnosed MDD using the Diagnostic and Statistical Manual of Mental Disorders, 4th edition [27], with reference to the Japanese version of the Patient Health Questionnaire-9 [28] and the Center for Epidemiological Scale-Depression [29]. Participants with both depressive and dementia symptoms were not diagnosed with MDD if the temporal order of events was not ascertainable.

### Vegetable and fruit consumption (exposure)

In 1995 and 2000, vegetable and fruit consumption was evaluated using a validated food frequency questionnaire [30, 31]. We used these data to calculate average vegetable and fruit consumption [32]. Participants reported the frequency and relative portion size of vegetables and fruits consumed in the preceding 12 months. The questionnaire included 29 vegetables (cabbage, Chinese radishes, komatsuna, broccoli, Chinese cabbage, leaf mustard, [Swiss] chard, spinach, Chinese chives, garland chrysanthemums, chingensai, mugwort, green pepper, carrots, tomatoes, pumpkins, tomato juice, onions, cucumbers, bean sprouts, snap beans, lettuce, bitter gourds, and loofah; and the following pickled vegetables: Chinese radishes, green leaf [Nozawana or leaf mustard], Chinese cabbage, cucumbers, and eggplant) and 17 fruits (papaya, apples, persimmons, strawberries, grapes, melons, watermelon, peaches, pears, kiwi fruit, pineapple, bananas, oranges, other citruses, pickled plums, 100% apple juice, and 100% orange juice). Flavonoid-rich fruits were defined as fruits containing a total flavonoid content of 50 mg/100 g [33]. We categorized each item into the following food groups: total vegetables and fruits, total vegetables, total fruits (including juice), total fruits excluding juice, and flavonoid-rich fruits (apples, pears, oranges, other citruses, grapes, and strawberries). Vegetable and fruit consumption was calculated by multiplying the frequency by the relative portion size. The food frequency questionnaire was previously shown to have acceptable validity and reproducibility [30, 31]. Spearman’s rank correlations between the food frequency questionnaire and independently evaluated dietary records were 0.22 in men and 0.32 in women for vegetable consumption and 0.41 in men and 0.23 in women for fruit consumption [31].

### Nutrient intakes (exposure)

Nutrient intakes obtained from vegetables and fruits were evaluated using the Fifth Revised and Enlarged Edition of the Standard Tables of Food Composition in Japan [34], again with averaging of data from 1995 and 2000. We included α-carotene, β-carotene, Vitamin C, Vitamin E, and folate. These measurements of nutrient intake were shown to have acceptable validity [35].

### Sociodemographic factors

We evaluated sociodemographic factors in 1995 that may be potential confounders. We included the following variables: age (continuous), sex (male or female), employment status (employed or unemployed), alcohol consumption (none, sometimes but not daily, or daily), current smoking (yes or no), and physical activity (none, sometimes but not weekly, or weekly).

### Statistical analysis

Statistical analyses were conducted using Stata 15 (Stata Corp LP; College Station, Texas). Multivariable logistic regression was used to examine the odds of MDD (outcome) according to quintiles of consumption of each food group (exposure) [36] and quartiles of nutrient intake (exposure) [37]. For flavonoid-rich fruits, we also studied individual fruit consumption. Grape and strawberry consumption was relatively low and was thus categorized into quartiles [36]. All vegetable and fruit consumption values were energy-adjusted to avoid confounding by total energy intake [38]. We fitted two regression models, an age- and sex-adjusted model and a fully adjusted model. We performed hierarchical adjustments for age, sex, employment status, alcohol consumption, current smoking, and physical activity. We evaluated linear trends by treating quantiles of vegetable and fruit consumption and nutrient intakes as a continuous variable. Further, we also evaluated odds ratios (ORs) of MDD per 10-g increase in the consumption of each group and flavonoid-rich fruit. We used bias-corrected and accelerated bootstrap confidence intervals (CIs) to obtain accurate information [39–41]. The size of the bootstrap sample was set at 1000 with 95% CIs [42].

## Results

Table 1 summarizes the demographics of participants in 1995 according to the lowest and highest quintiles of total vegetable and fruit, total fruit, and flavonoid-rich fruit consumption. Overall, the highest quintile groups were older, more likely to be female, less likely to be employed, and had fewer alcohol drinkers and smokers compared with the lowest quintile groups. Physical activity did not appear to be substantially different.

**Table 1:** Demographics of participants in 1995 according to lowest and highest quintiles of total vegetable and fruit, total fruit, and flavonoid-rich fruit consumption.

	No. (%)					
	Total vegetables and fruits		Total fruits		Flavonoid-rich fruits	
	Quintile 1	Quintile 5	Quintile 1	Quintile 5	Quintile 1	Quintile 5
Age, mean (SD)	56.9 (5.2)	59.0 (5.7)	57.6 (5.5)	58.4 (5.6)	57.3 (5.6)	58.7 (5.7)
Sex						
Female	64 (26.6)	209 (86.7)	66 (27.4)	198 (82.2)	54 (22.5)	199 (82.6)
Male	177 (73.4)	32 (13.3)	175 (72.6)	43 (17.8)	186 (77.5)	42 (17.4)
Employment status						
Employed	213 (88.4)	152 (63.1)	211 (87.6)	166 (68.9)	218 (90.8)	169 (70.1)
Unemployed	28 (11.6)	89 (36.9)	30 (12.5)	75 (31.1)	22 (9.2)	72 (29.9)
Alcohol consumption						
None	61 (25.4)	167 (69.3)	63 (26.3)	164 (68.1)	58 (24.3)	164 (68.1)
Sometimes but not daily	84 (35.0)	61 (25.3)	74 (30.8)	63 (26.1)	71 (29.7)	66 (27.4)
Daily	95 (39.6)	13 (5.4)	103 (42.9)	14 (5.8)	110 (46.0)	11 (4.6)
Current smoking						
Yes	91 (37.9)	8 (3.3)	89 (36.9)	13 (5.4)	99 (41.3)	8 (3.4)
No	149 (62.1)	232 (96.7)	152 (63.1)	226 (94.6)	141 (58.8)	231 (96.7)
Physical activity						
None	116 (50.2)	105 (45.3)	120 (52.6)	120 (51.3)	117 (51.5)	113 (48.3)
Sometimes but not weekly	62 (26.8)	63 (27.2)	63 (27.6)	56 (23.9)	61 (26.9)	62 (26.5)
Weekly	53 (22.9)	64 (27.6)	45 (19.7)	58 (24.8)	49 (21.6)	59 (25.2)

*SD* standard deviation.

Table 2 summarizes the odds of MDD by quintiles of vegetable and fruit consumption. In fully adjusted models, the highest quintile of total fruit consumption excluding juice and flavonoid-rich fruit consumption showed significantly decreased odds of MDD compared with the lowest quintile (OR = 0.34, 95% CI = 0.15–0.77; OR = 0.44, 95% CI = 0.20–0.97, respectively). Among flavonoid-rich fruits, strawberries showed significantly decreased odds of MDD in the highest quartile (OR = 0.37, 95% CI = 0.18–0.79, see Supplementary Table 1). No significant association was found for total vegetables and fruits, total vegetables, or total fruits. Odds of MDD per 10-g increase in consumption were significantly decreased for strawberries (OR = 0.76, 95% CI = 0.59–0.99) but not in any group, including the flavonoid-rich fruits group (Supplementary Table 2).

**Table 2:** Odds of major depressive disorder according to quintiles of vegetable and fruit consumption.

	Quintile 1	Quintile 2	Quintile 3	Quintile 4	Quintile 5	*P*_for trend_
Total vegetables and fruits						
Median intakes, g/d	279.7	423.8.4	541.1	680.9	922.3	–
No. of cases/controls	20/221	16/224	23/218	18/223	15/225	–
Age-, sex-adjusted model, OR (95% CI)	Ref.	0.72 (0.35, 1.50)	0.99 (0.52, 1.87)	0.71 (0.35, 1.44)	0.60 (0.30, 1.20)	0.22
Fully adjusted model, OR (95% CI)	Ref.	0.67 (0.32, 1.40)	0.91 (0.46, 1.81)	0.66 (0.31, 1.38)	0.55 (0.26, 1.18)	0.18
Total vegetables						
Median intake, g/d	143.3	221.9	294.3	369.8	529.1	–
No. of cases/controls	25/216	14/227	17/223	16/225	21/220	–
Age-, sex-adjusted model, OR (95% CI)	Ref.	0.49 (0.24, 1.02)	0.58 (0.30, 1.13)	0.50 (0.23, 1.07)	0.64 (0.32, 1.27)	0.25
Fully adjusted model, OR (95% CI)	Ref.	0.51 (0.23, 1.11)	0.57 (0.27, 1.18)	0.52 (0.25, 1.11)	0.60 (0.29, 1.25)	0.21
Total fruits						
Median intake, g/d	89.0	170.6	229.1	309.6	457.2	–
No. of cases/controls	22/219	18/222	18/223	19/222	16/225	–
Age-, sex-adjusted model, OR (95% CI)	Ref.	0.74 (0.38, 1.48)	0.68 (0.35, 1.31)	0.69 (0.35, 1.36)	0.57 (0.28, 1.14)	0.15
Fully adjusted model, OR (95% CI)	Ref.	0.74 (0.36, 1.52)	0.62 (0.31, 1.25)	0.65 (0.31, 1.36)	0.58 (0.27, 1.23)	0.18
Total fruits excluding juice						
Median intake, g/d	73.9	138.7	198.6	267.7	412.4	–
No. of cases/controls	24/217	16/225	19/221	22/219	12/229	–
Age-, sex-adjusted model, OR (95% CI)	Ref.	0.56 (0.28, 1.11)	0.63 (0.33, 1.19)	0.69 (0.37, 1.28)	**0.36 (0.17, 0.75)**	0.04
Fully adjusted model, OR (95% CI)	Ref.	0.50 (0.24, 1.04)	0.58 (0.29, 1.14)	0.59 (0.30, 1.15)	**0.34 (0.15, 0.77)**	0.04
Flavonoid-rich fruits						
Median intake, g/d	55.2	105.1	156.8	214.5	335.1	–
No. of cases/controls	22/218	20/221	17/225	19/221	15/226	–
Age-, sex-adjusted model, OR (95% CI)	Ref.	0.77 (0.39, 1.50)	0.60 (0.31, 1.16)	0.66 (0.34, 1.28)	0.49 (0.24, 1.01)	0.06
Fully adjusted model, OR (95% CI)	Ref.	0.67 (0.33, 1.35)	**0.49 (0.24, 0.996)**	0.54 (0.26, 1.12)	**0.44 (0.20, 0.97)**	0.05

*OR* odds ratio, *CI* confidence interval.

Fully adjusted models included age, sex, employment status, alcohol consumption, current smoking, and physical activity.

Bold values suggest significantly decreased odds.

Table 3 summarizes the odds of MDD according to quartiles of nutrient intake. No significant association was found for any nutrient.

**Table 3:** Odds of major depressive disorder according to quartiles of nutrient intake.

	Quartile 1	Quartile 2	Quartile 3	Quartile 4	*P*_for trend_
α-carotene					
Median intake, µg/d	295.0	565.2	871.3	1628.6	–
No. of cases/controls	27/273	22/280	18/283	26/275	–
Age-, sex-adjusted model, OR (95% CI)	Ref.	0.75 (0.40, 1.40)	0.58 (0.30, 1.12)	0.82 (0.43, 1.55)	0.41
Fully adjusted model, OR (95% CI)	Ref.	0.75 (0.40, 1.41)	0.59 (0.29, 1.19)	0.90 (0.47, 1.72)	0.67
β-carotene					
Median intake, µg/d	2101.4	3521.2	4901.1	7149.1	–
No. of cases/controls	26/274	24/278	18/282	25/277	–
Age-, sex-adjusted model, OR (95% CI)	Ref.	0.84 (0.46, 1.53)	0.56 (0.28, 1.12)	0.74 (0.39, 1.41)	0.24
Fully adjusted model, OR (95% CI)	Ref.	0.88 (0.47, 1.64)	0.55 (0.26, 1.17)	0.72 (0.36, 1.42)	0.21
Vitamin C					
Median intakes, mg/d	103.3	152.0	193.3	265.2	–
No. of cases/controls	24/276	25/276	23/278	21/281	–
Age-, sex-adjusted model, OR (95% CI)	Ref.	0.89 (0.52, 1.53)	0.76 (0.41, 1.38)	0.63 (0.33, 1.19)	0.16
Fully adjusted model, OR (95% CI)	Ref.	0.88 (0.49, 1.58)	0.73 (0.38, 1.40)	0.64 (0.33, 1.24)	0.21
Vitamin E					
Median intake, mg/d	6.2	8.1	9.7	11.8	–
No. of cases/controls	25/275	20/281	25/277	23/278	–
Age-, sex-adjusted model, OR (95% CI)	Ref.	0.67 (0.34, 1.32)	0.78 (0.41, 1.49)	0.65 (0.34, 1.24)	0.33
Fully adjusted model, OR (95% CI)	Ref.	0.60 (0.29, 1.25)	0.69 (0.36, 1.32)	0.55 (0.26, 1.18)	0.22
Folate					
Median intake, µg/d	337.7	432.9	518.4	654.8	–
No. of cases/controls	26/275	21/280	21/279	25/277	–
Age-, sex-adjusted model, OR (95% CI)	Ref.	0.70 (0.37, 1.32)	0.64 (0.33, 1.25)	0.72 (0.38, 1.35)	0.33
Fully adjusted model, OR (95% CI)	Ref.	0.73 (0.39, 1.39)	0.62 (0.31, 1.24)	0.68 (0.35, 1.34)	0.30

*OR* odds ratio, *CI* confidence interval.

Fully adjusted models included age, sex, employment status, alcohol consumption, current smoking, and physical activity.

## Discussion

As hypothesized, we found that consumption of flavonoid-rich fruits was inversely associated with a diagnosis of MDD. Similar findings were seen for the association of total fruit consumption excluding juice and MDD. These associations remained significant after adjusting for sociodemographic factors, including physical activity. These findings are consistent with past reports that fruit consumption had preventive effects on depression [7–12]. While a previous study showed that depression was inversely associated with the consumption of apples, pears, and citruses that contain flavonoids using data from older women, the present study is to our knowledge the first to demonstrate the antidepressive effect of overall flavonoid-rich fruits in a cohort that included both men and women.

We did not find antidepressive effects of vegetables, which is not consistent with previous studies [7–12]. The reason for this lack of association is unclear, but might reasonably be attributed to our study’s focus on MDD in later life, and longer follow-up period than previous studies [7–10]. Further, the data did not allow us to adjust for some potential confounders, e.g., psychosocial factors, that may confound the relationship between total vegetable consumption and MDD. Adjustments for other potential confounders are warranted.

While this study verified our hypothesis of an association between MDD and flavonoid-rich fruit consumption, an antidepressive effect was also found for total fruit consumption excluding juice. Considered together, the antidepressive effect might be attributed not to flavonoid-specific mechanisms [19] but rather to mechanisms involving fruit as a whole, e.g., reducing oxidative stress [13–17]. In contrast, total fruit consumption including juice produced non-significant results. Fruit juice is reported to have a lower antioxidant density than raw fruits [43], and may also have a higher glycemic index than raw fruits [44], which is suggested to be a risk factor for depression [8]. These mechanisms may partly explain the null findings when we included juice in the analyses. Indeed, a previous study showed that processed juice consumption was associated with an increased risk of depression [45]. Regarding nutrients, we did not find either protective or detrimental effects on MDD. However, this might have been caused by a lack of power to detect significant associations, considering that the highest quartile of the intakes of all nutrients showed decreased odds, albeit without significance. This lack of significance in nutrients in the present study warrants further exploration using a larger sample.

## Limitations

Some characteristics of the present study warrant cautious consideration. First, as mentioned above, we may not have adjusted for some potential confounders. Individuals with higher fruit consumption may have a healthier lifestyle. In the present study, such individuals may have positive factors other than dietary habits, e.g., psychosocial factors, which confounded the association between MDD and fruit consumption. Second, we did not have information on a diagnosis of MDD at baseline. On the other hand, we had information on a history of depression evaluated at mental health screening. To reduce the effect of reverse causation, we re-conducted the analysis with the exclusion of participants with a history of depression at mental health screening and found that the results were not changed (data not shown). Also, we used data for energy-adjusted fruit consumption to minimize the influence of appetite loss due to MDD at baseline. Nevertheless, the findings might have been affected by MDD at baseline. Third, although this is a population-based study, the sample comprised individuals aged 40–59 years at baseline, and the results might not be generalizable to populations with different characteristics. In the present study, we accounted for depression caused by dementia. Nevertheless, a sample comprising younger individuals may show a different association. Fourth, the sample size may not have provided sufficient power to detect the antidepressive effect of nutrients, e.g., Vitamin C. Similarly, the number of cases was limited, which restricted us to include only a limited number of covariates in the logistic models [46]. Finally, while our purpose was to evaluate the long-term effect of vegetable and fruit consumption on depression, some factors during the long-term follow-up might have affected the results. The long-term follow-up may have led to nonresponse of participants, causing selection bias. Further, although we used five-year average data, vegetable and fruit consumption might have been time-varying during the follow-up period, and our traditional models might have yielded biased estimates.

## Conclusions

The present study provides novel information on the association between MDD and flavonoid-rich fruit consumption, adjusted for sociodemographic confounders. Considered in the context of prior studies, these data further support the benefit of the consumption of fruit, including flavonoid-rich fruits. Future studies should employ a larger sample and adjustment for other potential confounders.

## Supplementary information

Supplementary information
