# Supplementary material for: Association between vegetable, fruit, and flavonoid-rich fruit consumption in midlife and major depressive disorder in later life: the JPHC Saku Mental Health Study
Source: Transl Psychiatry. 2022 Sep 26;12:412. doi: 10.1038/s41398-022-02166-8 (PMC9512814; doi:10.1038/s41398-022-02166-8)
Supplement: Supplementary file 1 — Supplementary information [file 41398_2022_2166_MOESM1_ESM.docx]

Supplementary Table 1. Odds of major depressive disorder according to quantiles of each flavonoid-rich fruit consumption. Note that grape and strawberry consumption was relatively small and thus categorized into quartiles.

|  | Quintile 1 | Quintile 2 | Quintile 3 | Quintile 4 | Quintile 5 | *P _for trend_* |
| --- | --- | --- | --- | --- | --- | --- |
| Apples and pears |  |  |  |  |  |  |
| Median intakes, g/d | 12.1 | 27.9 | 44.8 | 63.8 | 101.1 |  |
| No. of cases/controls | 20/220 | 20/221 | 20/222 | 15/226 | 18/222 |  |
| Age, sex-adjusted model, OR (95%CI) | Reference | 0.93  (0.47, 1.84) | 0.89  (0.45, 1.77) | 0.65  (0.31, 1.34 | 0.76  (0.38, 1.50) | 0.26 |
| Fully-adjusted model, OR (95%CI) | Reference | 0.92  (0.46, 1.82) | 0.87  (0.44, 1.73) | 0.56  (0.26, 1.21) | 0.70  (0.33, 1.48) | 0.19 |
| Oranges and other citruses |  |  |  |  |  |  |
| Median intakes, g/d | 24.6 | 54.8 | 82.0 | 117.9 | 211.0 |  |
| No. of cases/controls | 21/220 | 17/223 | 20/222 | 20/220 | 15/226 |  |
| Age, sex-adjusted model, OR (95%CI) | Reference | 0.74  (0.37, 1.49) | 0.80  (0.41, 1.59) | 0.79  (0.40, 1.57) | 0.56  (0.27, 1.18) | 0.19 |
| Fully-adjusted model, OR (95%CI) | Reference | 0.69  (0.33, 1.47) | 0.77  (0.39, 1.54) | 0.78  (0.39, 1.54) | 0.58  (0.26, 1.28) | 0.28 |
| Grapes |  |  |  |  |  |  |
| Median intakes, g/d | 1.4 | 5.4 | 10.9 | 24.5 |  |  |
| No. of cases/controls | 28/273 | 24/277 | 22/278 | 19/283 |  |  |
| Age, sex-adjusted model, OR (95%CI) | Reference | 0.84  (0.46, 1.53) | 0.73  (0.40, 1.34) | 0.60  (0.32, 1.11) |  | 0.09 |
| Fully-adjusted model, OR (95%CI) | Reference | 0.82  (0.45, 1.53) | 0.75  (0.40, 1.41) | 0.58  (0.29, 1.15) |  | 0.10 |
| Strawberries |  |  |  |  |  |  |
| Median intakes, g/d | 1.7 | 5.2 | 10.2 | 21.6 |  |  |
| No. of cases/controls | 28/272 | 28/274 | 22/279 | 15/286 |  |  |
| Age, sex-adjusted model, OR (95%CI) | Reference | 0.96  (0.54, 1.73) | 0.73  (0.40, 1.35) | **0.47**  **(0.24, 0.92)** |  | 0.02 |
| Fully-adjusted model, OR (95%CI) | Reference | 0.89  (0.49, 1.61) | 0.63  (0.33, 1.20) | **0.37**  **(0.18, 0.79)** |  | < 0.01 |

OR, odds ratio; CI, confidence interval

Fully adjusted models included age, sex, employment status, alcohol consumption, current smoking, and physical activity.

Supplementary Table 2. Odds of major depressive disorder per 10-g increase of the consumption of each group and flavonoid-rich fruit.

|  | OR (95% CI) |
| --- | --- |
| Total vegetables and fruits | 0.99 (0.98, 1.00) |
| Total vegetables | 0.99 (0.98, 1.01) |
| Total fruits | 0.99 (0.97, 1.00) |
| Total fruits excluding juice | 0.98 (0.96, 1.00) |
| Flavonoid-rich fruits | 0.98 (0.96, 1.01) |
| Apples and pears | 0.97 (0.91, 1.03) |
| Orange and other citruses | 0.99 (0.96, 1.02) |
| Grapes | 0.84 (0.69, 1.02) |
| Strawberries | **0.76 (0.59, 0.99)** |

OR, odds ratio; CI, confidence interval

The models adjusted for age, sex, employment status, alcohol consumption, current smoking, and physical activity.
